# Adapting an emergency department fall prevention intervention for persons living with dementia through patient, caregiver, and expert interviews

**DOI:** 10.1038/s41598-025-25290-z

**Published:** 2025-11-21

**Authors:** Elizabeth M. Goldberg, Caroline K. Tietbohl, Sandra García-Hernández, Megan Bounds, Jonathan Gomez Picazo, Hillary D. Lum

**Affiliations:** 1 University of Colorado Anschutz Medical Campus, 12631 E 17th Ave, 2nd Floor, Rm 2519, Aurora, CO 80045 USA; 2 Department of Emergency Medicine, University of Colorado Anschutz Medical Campus, Aurora, CO USA; 3 Department of Family Medicine, University of Colorado Anschutz Medical Campus, Aurora, CO USA; 4 Department of Hospital Medicine, University of Colorado Anschutz Medical Campus, Aurora, CO USA; 5 Department of Medicine, Division of Geriatrics, University of Colorado Anschutz Medical Campus, Aurora, CO USA

**Keywords:** Geriatrics, Public health

## Abstract

Study objective Although falls are up to three times more common in persons living with dementia (PLWD), limited fall prevention interventions exist for this population. Adapting promising interventions, such as the GAPcare intervention, which reduced fall-related ED visits by 66% and did not prolong ED length of stay, may address this need. In GAPcare patients receive pharmacy and physical therapy (PT) consultation to reduce modifiable risk factors for falls prior to ED discharge. In this qualitative descriptive study, we conducted semi-structured interviews with PLWD who recently visited the ED, their caregivers, and national experts in dementia care or ED operations to elicit perspectives on how GAPcare should be adapted for PLWD. Interviews were conducted in English and Spanish. We analyzed interviews using rapid qualitative analysis guided by Castro’s framework for adapting prevention interventions. We interviewed 7 patients, 2 caregivers, and 15 experts (5 physicians, 3 nurses, 3 PTs, 3 pharmacists, 1 PhD scientist). Participants strongly supported improved ED falls care for PLWD. They also indicated that tailoring at multiple levels (patient and caregiver, ED, and external factors, e.g., insurance status) would be required to support the complexities in PLWDs’ circumstances (e.g., living arrangements, income) and cognitive abilities. Participants suggested training of ED staff in dementia care and caregiver support. PLWD, caregivers, and experts in dementia care and ED operations are supportive of adapting our existing GAPcare intervention for PLWD. Early feedback from relevant informants guided GAPcareAD intervention refinement and fit with ED workflows.

**Supplementary Information:**

The online version contains supplementary material available at 10.1038/s41598-025-25290-z.

## Introduction

### Background and risk factors

Older adults with Alzheimer’s disease and related dementias (ADRD) fall up to three times more often than older adults with normal cognition.^1^ Mechanisms underlying fall risk in ADRD include decreased ability to navigate environmental demands, reduced gait speed, stride length, symmetry, and step regularity.^2–5^ Additionally, persons living with dementia (PLWD) frequently use anticholinergic and psychoactive medication that further increase fall risk.^6,7^ Cognitive impairment also limits engagement with interventions, making caregiver involvement essential for successful fall intervention delivery.

### Opportunity in the emergency department

Falls are the leading cause of ED visits among PLWD, accounting for nearly one in five encounters.^7–10^ An ED visit is a high-yield, under-engaged opportunity to address fall prevention.^11^ The Geriatric Acute and Post-Acute Fall Prevention Intervention (GAPcare) is an ED-based program in which pharmacists review medications and physical therapists assess mobility and balance, provide recommendations, and communicate these with outpatient clinicians. Notably, GAPcare addresses key fall risk factors in PLWD including impaired gait and balance, and high rates of anticholinergic and other psychoactive medications. In a prior randomized clinical trial, GAPcare achieved high patient and caregiver satisfaction,^12–14^ did not prolong ED length of stay,^14^ and resulted in 66% fewer 6-month fall-related ED visits compared to usual ED care.^15^ Due to its beneficial design, feasibility^14^, and efficacy^15^, GAPcare has the potential to rapidly disseminate, but adaptation for PLWD is necessary.

There are unique challenges of implementing interventions in the ED setting for PLWD that highlight why adaptation of GAPcare is necessary. ED care focuses on rapid evaluation and stabilization of acute conditions which is not typically aligned with the more chronic nature of complaints that PLWD have. PLWD often report more social needs which EDs may struggle to address due to limited resources (e.g., no social work services, limited training of ED clinical staff in how to address these). EDs also do not routinely screen for dementia and thus underrecognize how much dementia may play a role in the patient’s presentation, ED diagnosis, or ability to adhere to follow-up. PLWD are also more likely to reside in facilities (e.g., memory care) and require coordination with facility staff to make changes to treatment plans.

### Goals of this investigation

Because most ED-based fall studies have excluded PLWD,^11^ existing fall prevention interventions have not been designed to effectively address ADRD-specific fall risk factors and prevent fall-related ED visits. As a result, evidence-based interventions implemented for the general population in current clinical practice have not improved fall-related outcomes among PLWD. To address this gap, we sought to adapt GAPcare for use in PLWD (GAPcareAD). This study engaged patients, caregivers, and experts in dementia care and ED operations to identify intervention preferences and develop a prototype protocol for GAPcareAD, GAPcare adapted for dementia, ahead of a planned randomized controlled trial. By engaging key informants in adaptation efforts, we can increase the effectiveness of the planned intervention.^16–18^ Our long-term goal is to prevent falls and their sequelae among PLWD, downstream healthcare visits and institutionalization, which reduce healthy days at home and quality of life.^19,20^.

## Materials and methods

### Study design

**Fig. 1:**
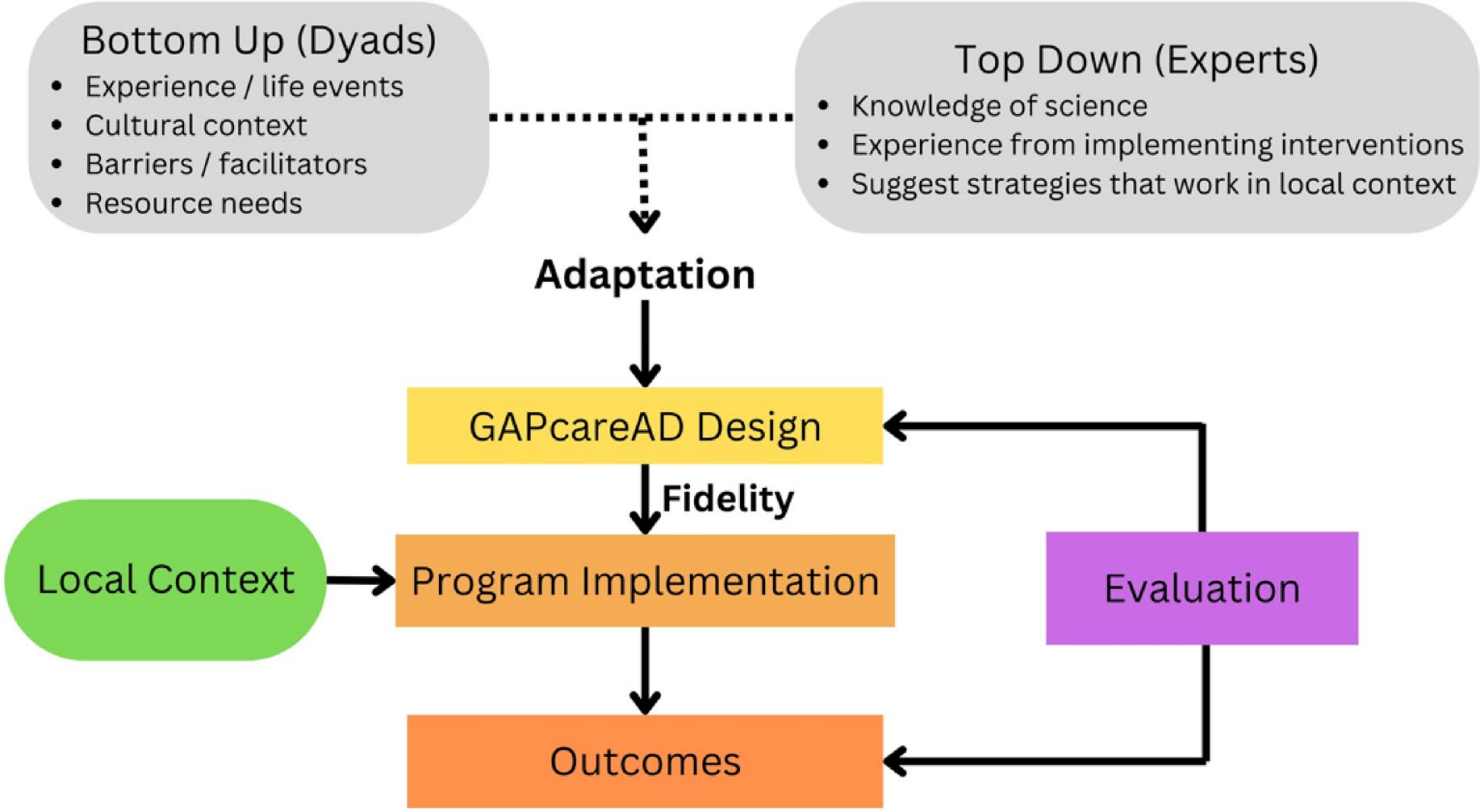
Castro’s framework for adapting interventions with fidelity, modified for GAPcareAD. This paper focuses on adapting the intervention to inform the design. In the future we will test its outcomes

We used the framework by Castro^17^ for adapting prevention interventions with fidelity (see Fig. 1) to inform our study design. This framework emphasizes community participation to enhance outcomes (bottom-up approach). Patients and caregivers from the community contribute valuable lived experience that can inform feasibility of the intervention in real-world settings. Experts contribute knowledge of science and experience implementing interventions to enhance adaptation (top-down approach). The adapted intervention (GAPcareAD) is then implemented with fidelity to the core components which are thought to be essential to the efficacy of the intervention while making refinements to ensure it is optimally compatible with the local context, e.g., community EDs. Outcomes are measured and contribute to the evaluation and further improvement of the intervention. The consolidated criteria for reporting qualitative studies (COREQ)^21^ standards was used to report findings for this qualitative study.

### Setting and participants

Purposive sampling was used to recruit a) PLWD and their caregivers, and b) national experts in dementia care and ED operations. Patients and caregivers were recruited from a quaternary care ED with 115,000 annual ED visits. Eligibility criteria for patients included community-dwelling (non-institutionalized), ≥ 65 years old, capacity to consent, and speak either English or Spanish. We included Spanish-speakers because 1 in 3 older adults who present to our study site are Hispanic. We followed our institutional guidance to assess capacity to consent. Patients were given the opportunity to demonstrate functional ability to understand the purpose of the research study. We used teach-back questions to assess patient understanding of study procedures, risks, benefits, and their rights, and each patient had three opportunities to correctly answer the question before being excluded for lacking capacity. Eligible patients had to have mild cognitive impairment (MCI) or dementia on their problem list or score > 2 on the AD-8 Dementia Screening Interview.^22,23^ The patient identified a caregiver to participate. We approached potentially eligible patients in the ED after electronic health record (EHR) review. We held interviews individually or as a dyad, during the ED visit or afterwards. Our discussions with implementation experts in preparation for this study suggested that PLWD and their caregivers would be most likely to engage in the study if we offered different interview modes (e.g., in person, remote) and flexibility in terms of single vs. dyadic interviews. A master’s level qualitative analyst performed the interviews under the guidance of a qualitative PhD scientist (CT).

We recruited national experts across various roles (ED-based nurses, advanced practice providers, physicians, pharmacists, PTs, PhD scientists) and areas of expertise (dementia, caregiving, ED operations) to provide perspectives. The GAPcare intervention is an intervention that requires engagement from ED nurses, physical therapists, and pharmacists; thus, we included these professionals. Additionally, we included geriatric EM trained physicians and scientists because they commonly design research studies that involve this patient population and have intimate knowledge of barriers and facilitators that may be encountered in the implementation of this work.

### Sample size considerations

We aimed to continue recruitment until thematic saturation was achieved, consistent with accepted qualitative standards^24^ We balanced the need for data saturation with pragmatic considerations of time and participant burden in clinical settings. Our planned sample size was consistent with prior qualitative studies among older ED patients. n = 5–15.^25,26^.

### Data collection

We developed separate semi-structured interview guides for interviews with patients individually, caregivers individually, patient-caregiver dyads, and national experts (see guides in Supplementary Information). All interview guides included questions regarding perceptions of the GAPcare intervention, preferred adaptations, barriers and facilitators to implementation, and logistics of implementation. We created interview guides based on our clinical expertise of fall-risk factors and our prior interviews with participants in our fall prevention clinical trial which recruited ED patients after a fall.^12,27^ Interviews were piloted within the study team and refined by our qualitative lead (CT).

A bilingual master’s-level research analyst with qualitative training (SG) and another trained research assistant (MB) conducted interviews with guidance from the principal investigator (EG) who has graduate training in qualitative research, and a qualitative PhD methodologist (CT). Interviews were 30–45 min and conducted via telephone, video, or in person depending on participant preference and availability. Participants received a gift card after the interview. Interviews were recorded and professionally transcribed. All study participants provided informed consent. The study protocol was approved by the Colorado Multiple Institutional Review Board (COMIRB). All methods were performed in accordance with COMIRB guidelines and regulations.

### Data analysis

We used a rapid qualitative analysis approach to analyze all interviews. This approach is appropriate for implementation research that seeks to generate recommendations within a short timeframe.^28^ First, two team members (CT, SG) developed a templated summary form that included domains informed by the Castro framework, the interview guide, and preliminary themes that emerged inductively during data collection. Each analytic team member independently read and completed a summary form for one interview, then met to reconcile differences and refine the summary form. This process was repeated a second time to establish consistency. The two analytic team members then reviewed the remaining transcripts and completed a summary form for each. Next, a matrix analysis was conducted to identify themes across interviews.^29^ Summary form data was entered into a matrix within Excel (Microsoft 365; 2024) and themes were identified by comparing responses within and across interviews, writing analytic memos to summarize findings, and having regular team discussions. Investigator triangulation (multiple investigators with multiple areas of expertise) was used to establish the trustworthiness of our findings.^30^.

## Results

From August 2023 to April 2024, we interviewed 7 patients and 2 caregivers (1 dyadic interview, 7 individual interviews), and 15 experts (5 physicians, 3 nurses, 3 PT, 3 pharmacists, 1 PhD scientist). Seventeen of 23 interviewees were women. Patient and caregiver demographics are summarized in Table 1, and expert characteristics are in Table 2.

**Table 1:** Patient and caregiver characteristics

Characteristic	Group	Caregiver, n % (N = 2)	Patient, n % (N = 7)
Age	60–69	100 (2)	28.5 (2)
	70–79	0(0)	28.5 (2)
	80–89	0(0)	28.5 (2)
	89 +	0(0)	14.2 (1)
Sex	Male	50 (1)	42.8 (3)
	Female	50 (1)	57.1 (4)
Race	White or Caucasian	100 (2)	85.7 (6)
	Black or African American	0(0)	14.2 (1)
Ethnicity	Non-Hispanic	100 (2)	100 (7)
	Hispanic	0 (0)	0 (0)
Preferred language	English	100 (2)	100.0 (7)
Education level obtained	Any Post-Graduate Training	50 (1)	28.5 (2)
	College Graduate	50 (1)	14.2 (1)
	Some College	0 (0)	28.5 (2)
	High School Graduate or GED Certificate	0 (0)	14.2 (1)
	Unknown	0 (0)	14.2 (1)
AD-8 Scores, median (IQR)		N/A	4.0 (3.0–5.0)

**Table 2:** Expert characteristics (n = 15)

Characteristics	Group	n, %
Age	30–39	53.3 (8)
	40–49	33.3 (5)
	50–59	13.3 (2)
Sex	Male	20.0 (3)
	Female	73.3 (11)
	Unknown	6.6 (1)
Race	White or Caucasian	80.0 (12)
	Black or African American	6.6 (1)
	Multiracial	6.6 (1)
	Other	6.6 (1)
Profession	Physician	33.3 (5)
	Physical therapist	20.0 (3)
	Pharmacist	20.0 (3)
	Nurse	20.0 (3)
	PhD scientist	6.6 (1)
Specialty	Geriatrics	53.3 (8)
	Emergency medicine	26.6 (4)
	Adult gerontology emergency services	6.6 (1)
	Public health/Aging	6.6 (1)
	Unknown	6.6 (1)
Years in medicine	0	40.0 (6)
	< 5	13.3 (2)
	5–10	20.0 (3)
	10–20	20.0 (3)
	> 20	6.6 (1)
Involvement in dementia or caregiving research	No involvement	13.3 (2)
	Little involvement	26.6 (4)
	Some involvement	33.3 (5)
	Very involved	26.6 (4)

Interviews revealed that patients, caregivers, and experts strongly supported improved ED falls care for PLWD. However, participants reported that tailoring at multiple levels (patient, ED, and external environment, e.g., patient’s insurance coverage) would be required for the intervention to succeed given the complexities in patients’ circumstances and cognitive abilities. These themes are summarized in Table 3.

**Table 3:** Summary of themes for adapting the GAPcare intervention with supporting quotes

Theme	Description	Quote
Patient tailoring	Importance of recognizing the patient’s stage of dementia and tailoring communication appropriately Balancing reliance on caregivers for information while still engaging and focusing on the patient Education for caregivers of PLWD regarding the pharmacist’s role and the difference between GAPcare PT and other PT	“It can be a very, very complex and multifactorial picture that may not be really possible to solve all the pieces of the puzzle with limited time, with limited history from the patient with cognitive dysfunction unless patient has a very, very supportive caregiver who knows everything with all the details and is available in the emergency department and taking the lead from the assessment and the evaluation and planning, that’s taking a major role for helping the emergency department physician.” (Expert #308) “Sometimes, I think if a patient falls, and especially if it’s a patient who has dementia, and whenever they’re being questioned, if they seem confused, I feel like sometimes, that can be an escalation to admit that patient versus noticing at baseline, "Oh, okay, this patient has dementia." (Expert #315) On what reminders of the action plan would be helpful after the ED visit for those with more advanced dementia, one patient stated: “Let’s see. If I had problems remembering—that’s the best thing I can think of. It would probably be that phone call or some people—maybe an alarm or something.” (Patient #106)
ED tailoring	Medication reconciliation is challenging because clinicians must rely on caregivers or EHR, but neither may be accurate Developing clear steps and roles for medication reconciliation is needed to deliver GAPcare to patients with ADRD The timing of intervention delivery should be tailored to each ED, including which personnel would be responsible for implementation depending on the time of the patient’s admission to the ED	“Yeah, so he’s not aware of what he’s taking, and I have not done a very good job of keeping that at my fingertips. I think I put an updated list in my packet in the car for next time, but emergency trips, by definition, catch you off guard, so that was hard. I didn’t have a complete list and dosage and all that stuff.” (Caregiver #201) “If you don’t have the coverage on the weekend, how do we capture this patient that really would benefit? …[is this] so automated that [it works] even if it doesn’t happen while the patient’s in the emergency department? Because we don’t have the resources for this specific intervention, ’cause we don’t have 24/7 physical therapy, does this mean we’re gonna have to put more people in our observation area to wait for the GAPcare intervention, increasing our length of stay, increasing the number of patients that are physically in the department for longer?” (Expert #303)
External environment tailoring	The patient’s living circumstances will impact delivery of GAPcare and may require specific processes (e.g., living at an assisted living facility) The patient’s insurance coverage may also impact delivery of GAPcare and could require tailoring	“One thing I think that would need to happen is there would need to be good communication with patients who live in a facility to make sure that if a medication is identified as part of the fall, and it’s stopped, that the facility gets communication from the ER to say to stop it. It has to be a—usually it can’t be a verbal order. It has to be a written or faxed order. That would be something that would be really important to think about. How is that gonna be operationalized?” (Expert #302) “Yeah, I think it’s a good approach, and I hope the insurance companies support it ’cause I think that would be a big thing. With my mom, if they said, ‘Well, the insurance company won’t pay for this,’ it—we don’t have a lot of resources so for her to do that.” (Caregiver #202)

### Acceptability of GAPcare for PLWD

Experts, patients, and caregivers all expressed support for GAPcare and reported that the intervention would be beneficial in the ED. Most experts worked in settings without a unified approach to fall prevention, though many reported that this support is needed because falls are common among PLWD. Few patients had received any fall assessment or were aware of the reason for their fall.

*“Well, I have a weak knee and a weak hip, which I’m sure a physical therapist would be good for.” (Patient #51)*

Additionally, experts described that the ED was an appropriate time to intervene because patients may be more motivated to invest in prevention following a fall. A few patients and caregivers had pieced together the reason for the patient’s fall on their own, but those who did not reported that they would have wanted to learn more about what they could do differently in the future. Across interviewees, participants felt that receiving additional support would be critical for PLWD and caregivers to implement any recommendations at home:

*“[GAPcareAD] would be helpful because when you’re in the emergency room, there’s just so much different information coming at you that it’s hard to remember three days and a week later what you’re supposed to be doing, what’s recommended or questions about trying to implement.” (Caregiver #201)*

However, all interviewees described some challenges to delivering GAPcareAD in the ED for PLWD. The following sections explain adaptations that participants identified to tailor GAPcareAD at the patient level, ED level, and external environment level.

### Patient tailoring

Across participants, an important aspect of tailoring GAPcare for PLWD was accounting for the patient’s level of cognition and determining the level of support they receive or need from a caregiver. Yet, experts noted that it can be difficult to identify dementia among older patients without a knowledgeable caregiver present. Patients may be confused in the ED, and clinicians may attribute these symptoms to the acute reason for the visit rather than to underlying dementia. As a result, it can be difficult to elicit accurate information about the patient’s history and to determine treatment – including recommendations around fall prevention.

*“I think it’s…harder to communicate with those patients, particularly in the absence of reliable care partners because you don’t know the severity of their dementia. Oftentimes you don’t recognize they even have dementia. Sixty percent of our patients that have cognitive impairment if you formally test them, flow through [the] ED undetected…it’s gonna be harder to communicate with those folks both in terms of have they had a fall, do they remember to tell you about the fall, and do they remember the instructions you give them for post ED management with physical therapy, with medication reconciliation with pharmacy, with follow-up with a falls clinic if you have one.” (Expert #304)*

To address this issue, participants emphasized the importance of having a supportive, involved caregiver present to communicate with clinicians in the ED. While multiple patients felt comfortable getting around, scheduling appointments, and talking to clinicians on their own, caregivers and experts felt that patients often struggle to recall details related to medications and health behaviors. As some caregivers explained, their involvement was even more important for patients with greater limitations:

*“The other issue with my mom is her communication skills. She had a stroke several years ago, and so she can communicate, but she has struggles sometimes finding her words [so] it’s difficult to get her to answer a question.” (Caregiver #202)*

Experts also felt that implementing GAPcareAD would require additional education about dementia, such as communication training.

*“I think the other piece is that a lot of providers are deeply uncomfortable taking care of older adults [laughter] and particularly older adults with dementia. It’s just really under trained in our medical training… Not enough people get exposure to that patient population in medical school or residency or whatever training route they went, and so, people just are like, ‘I don’t know what to do.’” (Expert #313)*

Similarly, patients and caregivers also expressed a need for more education around what the extra support provided by GAPcareAD would entail. For example, some caregivers conveyed skepticism about how helpful PT would be given their prior experiences working with PT to learn exercises at home. Education around how GAPcare PT–tailored towards fall prevention, strength, balance, and correct assistive device use—differs from the PT patients and caregivers may be more familiar with could help engage them in the intervention.

### ED tailoring

Interviewees from all three participant groups described challenges around medication reconciliation. Caregivers reported that PLWD would not be able to manage their own medications, but also had difficulty keeping up with patient medication lists themselves.

*“I’m just gonna be honest. I don’t trust medication reviews that I don’t do. I just see they’re inaccurate a lot of the time because the family doesn’t necessarily know if they don’t have the bottles with them. If the patients have a PCP somewhere else and [EHR] doesn’t have their accurate medication list, which [EHR] is not always accurate anyway, then I think it’ll be difficult to know for sure what medications are on it unless the pharmacy fill history is reviewed.” (Expert #302)*

Participants were open to working with a pharmacist to assist with medication reconciliation, but many expressed a need for more education around the pharmacist’s role in the intervention. Most interviewees felt that it was important for the pharmacist to coordinate with the patient’s PCP to ensure that any medication changes were appropriate, however, this coordination may depend on the ED’s resources and personnel involved in delivering the intervention.

Additionally, participants explained that adapting GAPcare for PLWD would also depend on the timing of its delivery in the ED. For example, experts described that most older patients get injured in the middle of the night, raising questions about whether the intervention would be offered at all hours and what personnel would be available. Experts acknowledged that this may differ depending on the ED; some reported that the ED physician would need to reconcile medications, while others expressed that the pharmacist should manage this task at their ED. For patients and caregivers, the distribution of labor among varying personnel was less important than ensuring sufficient time to carry out the intervention thoroughly.

*“I just feel that sometimes things move so fast in the ER that people, the elderly people that do the slip and falls need to have time to answer their questions properly. That needs to be somebody that they’re gonna see there to take the time to answer the questions and feed the rest of the team the information.” (Patient #38)*

### External environment tailoring

Finally, participants explained that the patient’s external environment and circumstances should also be considered when tailoring GAPcare for PLWD. For example, some participants reported that coordinating and implementing care for patients who reside in assisted living facilities (ALFs) can be challenging because the ALF staff may not implement any changes unless they receive a written order (e.g., from the patient’s PCP). Thus, follow up care for patients who live in ALFs can be delayed and potentially contribute to avoidable rehospitalization. As one expert explained, this can cause patients to continue “getting that [medication], even if the ER stops it” (Expert #302).

Participants also expressed the need to clarify the patient’s insurance coverage when tailoring GAPcare for PLWD. For many patients, this information may need to be coordinated with the primary caregiver to determine what services are covered, whether the patient requires any additional support devices, and whether they should expect copays. Experts felt that for PLWD, the intervention needed to be delivered at least partially at home for patients to benefit, and that this care could be billed in a cost-effective manner. As one expert noted:

*“Honestly, somebody needs to go into the person’s home in my opinion, and that person could be a physical therapist and could bill for that service as under Medicare part B. I do think that what you do in the ED, with all the chaos of everything else going on, is helpful, but it might not stick with people, or the benefits might not be fully realized if we don’t get to that person early after their discharge and make sure they can implement the things that we’ve suggested to them.” (Expert #317)*

Caregivers and patients reported that receiving additional support via home visits would be welcome. However, even though caregiver involvement was identified as an essential component of adapting GAPcare for PLWD, caregivers explained that their role is determined by the patient’s preferences around how involved they want caregivers to be in their care. Identification and coordination with family caregivers who live with the patient can also be challenging depending on the caregiver’s availability and level of burnout.

Following recommendations made by our participants and informed by the Castro framework, we adapted several elements of GAPcare relevant to staff training, intervention delivery, supporting patients and caregiver participants, and enhancing recruitability and retention (Table 4).

**Table 4:** Adapted GAPcare intervention, GAPcareAD, incorporating feedback

Category	Lesson learned	GAPcareAD adaptations and improvements
Staff training	Medication review challenging in ADRD; care not tailored for PLWD and caregiver needs	Use dispense records, lists, caregiver reports to get accurate medication lists; targeted staff training in ADRD and caregiving; consider EHR alert to help staff identify ADRD
Interventionalist training	ED clinical staff, PTs, pharmacist feel unprepared to treat persons living with dementia	Provide tailored, engaging training in dementia care specific to staff roles, e.g., PTs provide simple instructions on how to use assistive device to both patient and caregiver
Assessment burden	Caregivers have competing demands, making follow-up scheduling difficult	Reduce burdensome surveys by using EHR and Medicare data to track healthcare utilization and action plan uptake
Intervention delivery	Study documents often lost due to multiple transitions in care, recommendations forgotten	Purposely engage caregivers; send welcome letter to remind participants of study milestones and contacts to study staff; include pharmacy and PT recommendations in the welcome letter; send welcome letter to both patient and caregiver
Intervention delivery	Tailor to stage of ADRD; make cognitive adaptations	Simplify language, minimize amount of information presented, use multi-input education–reading, auditory, visual–to explain intervention elements
Follow-up	Patients and caregivers may forget about the study and PT/pharmacy recommendations	Orient participants to the study at follow-up calls and use the same staff member, when possible, to do follow-ups; provide reminders of recommendations made to reduce falls by providing written recommendations during the ED visit, sending mailings after the ED visit, communicating with PCPs and facilities
Supporting participants	Some patients may have social determinants of health that reduce adherence	Provide resources in form of linkage to care with state elderly affairs and community-based organizations; screening for social determinants of health at follow-up visits
Retention	Caregiver acts as study gatekeeper and overwhelmed by burdensome follow-up	Educate caregivers on study benefit, add caregiver education and compensation for involvement

### Limitations

We followed recommendations to include key informants early and throughout the intervention adaptation process,^31^ however, we acknowledge some limitations. Patients and caregivers were recruited from a single academic ED in the Mountain West, and their experiences and perspectives may differ from individuals living in other regions. Similarly, all caregivers were adult children; including diverse types of caregivers such as spouses or friends may have yielded different perspectives. Overall, few caregivers were included citing lack of interest or time. Although the caregivers we interviewed did not raise topics that differed from patients/experts around the intervention, it is possible that more interviews could have elaborated on our findings further. Additionally, although analysis was performed by a team of researchers from different disciplines (geriatrics, sociology, emergency care), all individuals have inherent biases that may have influenced the interpretation of results.

## Discussion

The purpose of our study was to adapt our existing fall prevention intervention guided by the Castro framework for a new population before clinical trial testing. Qualitative feedback from key informants in the community and nationally generated several suggestions that informed the adapted protocol. While our interview guides focused on adapting specific components of an existing fall prevention intervention, several lessons learned could be adopted to improve ED care for PLWD.

One key theme was that communication between ED clinical staff and ED patients and caregivers, and between ED clinicians and outside facilities needs to be prioritized. Several steps should be taken to improve communication, (1) older patients should be assessed for dementia, including severity of dementia, to guide tailored communication, (2) patient engagement should be prioritized while also gathering and sharing information with the caregiver, (3) caregivers should be identified and included in the patient’s clinical care, where possible, (4) clinical recommendations should be shared with facility staff to optimize uptake of post-ED care plans, and (5) ED clinical staff should receive specific training in dementia care, so they can achieve these communication goals. While these suggestions may seem difficult in the context of typical ED demands, there are advantages to aligning ED care with PLWD and caregiver needs. Potential benefits include improvement in the patient experience, optimized care transitions, and reduced adverse events for PLWD.

Another key theme was that strategies to obtain accurate health information on ED patients with impaired cognition are lacking. This is partly due to the urgency of the ED visit, which makes it difficult for patients/caregivers to collect necessary information beforehand, and in part due to the cognitive difficulties inherent in dementia. In particular, caregivers and experts expressed concerns over medication reconciliation. An accurate medication list is an important prerequisite to identifying medication-related reasons for the fall and modifying fall-risk increasing medication to improve fall risk, a core component of the GAPcare intervention. Some solutions exist; more accurate lists can be obtained when querying the caregiver in addition to the patient, using medication lists, and pharmacy dispense records.^32^ Although many ED clinical staff play a role in medication reconciliation, interviewees familiar with ED operations cited that it will be important to assign roles for this task, and ED pharmacists may be best equipped to perform these reconciliations.

Another important theme was the tension between offering a brief intervention that could be easily implemented in resource-limited EDs versus a more complex, comprehensive intervention that would require longer to implement but may offer the most benefit. In the prior GAPcare clinical trials, our team gained experience with optimizing the implementation of a multicomponent intervention for older ED patients.^14,15^ We paid specific attention to timing intervention components so they would minimally interfere with usual ED care, while also offering a benefit to patients in recognizing their fall risk factors and addressing them. Pharmacy and PT consults took a median of 20 min each, and ED length of stay was not prolonged for intervention participants.^33^ However, because PLWD often have greater communication challenges and coordination needs, we recognize the need to simplify the intervention and provide more time for education, explanations and reinforcement, and coordination of care. As some interviewees suggested, this may require GAPcareAD to have a post-ED component, whereby clinical staff knowledgeable of care transition challenges in dementia reinforce recommendations made by the pharmacist and PT. Balancing the suggestions to enrich the intervention, while also being mindful of ED staff burden and efficiency needs, will be an important challenge to overcome during implementation. Incentives that support improved dementia care in the ED, such as quality metrics or billing codes relevant to improved ED dementia care could help support these prevention efforts.

Other research teams have adapted interventions for dementia patients or caregivers, and found similar themes. These studies found that strengthening educational messages to enhance comprehension and uptake are important, and they found a perceived necessity of caregiver involvement in healthcare interactions.^34,35^ They also found that interventions may be more ideally suited for PLWD who have not progressed to the advanced stage.^35^ Patients with varying stages of dementia will be included in GAPcareAD, but we plan to evaluate differences in efficacy by ADRD stage.

## Conclusion

ED patients with impaired cognition, associated caregivers, and experts in dementia and ED operations and research strongly support adapting an ED fall prevention intervention for dementia, and suggest that tailoring to the individual patient, ED environment, and the ED’s external environment is important to ensure success of the adapted intervention. This work has informed the adaptation of GAPcare to ensure it meets the unique needs of PLWD and their caregivers who are assessed in the ED for falls.

## Supplementary Information

Supplementary Information.

## Data Availability

As the data collected is qualitative and contains participant identifiers, the data will not be publicly available. The point of contact is Dr. Elizabeth Goldberg, MD, ScM.
