# Supplementary material for: Adapting an emergency department fall prevention intervention for persons living with dementia through patient, caregiver, and expert interviews
Source: Sci Rep. 2025 Nov 21;15:41399. doi: 10.1038/s41598-025-25290-z (PMC12638784; doi:10.1038/s41598-025-25290-z)
Supplement: Supplementary file 1 — Supplementary Information. [file 41598_2025_25290_MOESM1_ESM.pdf]

Supplementary Table 1. Interview guide for Dyads, Caregiver Only, and Experts

Aim 1 Interview Guide for Persons with Cognitive Impairment and their Caregiver

This interview agenda is a guide for the interviewers, but not a rigid script. We have added intent statements explaining the goals of a particular line of questioning for each major block of questions. Facilitators will use the agenda to ensure that all topic areas and probes are covered in the way we intended them to be covered. Questions may be added or deleted or altered in subsequent interviews as dictated by previously collected data (e.g. data saturation, emergent topics/themes) and in accordance with good qualitative methodology.

Intent: The intent of these interviews is to determine the perspectives of patients after an emergency department visit for a fall to elicit barriers/facilitators, and ideas for how to facilitate post-fall care. We will review the participants' characteristics prior to the interview: age, race/ethnicity, living situation, relationship to the caregiver. We will collect participants' reflections about the GAPcare intervention. If they were not in the GAPcare intervention, they will receive a flyer to review about it. Questions can be answered by either the person with cognitive impairment or their caregiver, however we have a separate guide for the caregiver.

Semi-structured interview protocol

Introduction:

Hello, my name is [name], and I am a [role] at the University of Colorado. Thank you for agreeing to meet with me today. We will be talking about [patient name]'s recent visit to the emergency department to learn more about how to improve care for patients after a fall. This interview should last about 30 minutes.

I'm just going to go over a few points from the consent form before we begin.

- You are being asked to be in this research study because [patient name] recently had a fall and visited the emergency department on [date].
- Every effort will be made to protect your privacy and confidentiality by not using your name on any research materials only a study ID will be used. All results will be submitted in aggregate form only and no names will be disclosed, so your responses will not be identifiable.
- This interview is voluntary. There are no right or wrong answers, and you may choose to answer or not answer any questions.
- If you have questions about the study, you can contact Dr. Elizabeth Goldberg at elizabeth.goldberg@cuanschutz.edu.
- You may have questions about your rights as someone in this study. If you have questions, you can call COMIRB (the responsible Institutional Review Board) at (303) 724-1055.
- I want to be sure I do not miss anything that you say, so I am asking your permission to record our conversation today. Do I have your permission to record?
- If you are ready, we will get started with the questions.

[State date and participant ID for the recording]

Barriers/Facilitators

Intent: The intent of this section is to understand participants' reactions to the planned GAPcare intervention, so as to understand what aspects of the intervention may need to be refined or specifically tailored to persons with cognitive impairment. Please provide patients and/or caregiver with the GAPcare handout for their review before starting questions.

1. The reason we are doing this study is because we want help patients who have had a fall and visit the emergency department. This handout shows the GAPcare intervention, which is extra care that patients like you would get in the emergency department after having a fall. As you can see on the handout, with GAPcare a team of professionals, including physical therapists and pharmacists, would evaluate you while you're waiting in the emergency department and then you would talk to this team to come up with a plan for how to prevent falls at home. What are your initial reactions to getting a special fall evaluation and treatment plan while you're waiting in the emergency department?
2. Tell me how you think it would feel to talk to a physical therapist to figure out the reasons for <patient name's> fall and come up with a plan to support their care after they return home.

a. What about talking to a pharmacist about your current medications and how they could be changed to help prevent falls?

3. What concerns do you have about <patient's name> receiving care from a physical therapist, if any? (probe for Rationale)
4. Are there additional considerations that are unique to your culture or family that you think might impact the [patient's name] experience with GAPcare? (e.g., willingness to be involved, perceptions of receiving help from the healthcare team, ED procedures, follow-up procedures).

a. How do you think possible home treatments or remedies may work into this plan?

Acceptability

Intent: The intent of this section is to ask participants to describe their acceptability of the planned intervention. This section also assesses what support they need at home after a fall.

5. In your experience, would <patient's name> struggle with any of the following parts of GAPcare? These are some of the things they would have to do **in the emergency department**:

a. Speak with a pharmacist and respond to questions about their medication (e.g. what are their current medications, are they taking them, are they experiencing side effects, would they be open to modifying or stopping them with their doctor's permission?)

b. Participate in mobility exercises like getting up from a chair or walking with assistance with a physical therapist (probe for Rationale)

6. After visiting the emergency department, the plans that patients take home to help prevent falls can include a few different things. Now, I'd like to ask you about what it might be like for **you to support** <patient's name> to do each of these activities **at home**. For each of these items we'd like to know if [patient's name] can do it alone or if they have assistance.

a. Arrange a follow-up appointment with their primary care clinician. At the appointment, would they/you be able to talk to their doctor about the recommendations the pharmacist made in the hospital? (probe for Rationale)

b. Arrange an appointment with a physical therapist in an outpatient office or at home? (probe for Rationale)

c. Work with a physical therapist and do exercises at home and/or outpatient offices. What would it be like for you to help <patient's name> do that?

d. Help them to remove clutter and hazards away from areas where they walk?

e. Help them to use their cane/walker consistently when they are moving around the home?

f. Help them wear supportive shoe wear whenever they leave the bed?

g. Do you have any concerns about supporting <patient's name> at home after their fall?

h. What type of support would you need to help make sure <patient's name> is following recommendations like these, if any?

i. Would scheduled phone calls after the ED visit at 72 hours and one week help?

Adaptations (Cognitive/Cultural)

Intent: In order to better understand how to modify the GAPcare intervention to make it optimal for people with cognitive impairment and caregivers, especially those with diverse cultural traditions, we will ask questions about desired adaptations.

7. Many patients find it helpful to get reminders about what they are supposed to do once they get home. What do you think would help you remember to follow recommendations for preventing falls at home? (E.g., Phone call reminders/magnet for fridge/follow-up letter/app or telehealth reminders/text messages)

a. What reminders would be helpful to you if you were helping <patient's name> follow a fall prevention plan?

b. For caregivers: If you typically do not attend ED visits with <patient's name> what type of handoff would you want to receive on the plan so you could act on it? (e.g. checklist, phone call, written document)

8. Based on what we have talked about so far, what role do you think the caregiver should play in the intervention? (e.g., accompanying patient to PT visits or to PCP visits to ensure GAPcare medication recommendations are incorporated, working with the patient to make medication changes to over the counter medications, etc.)

Implementability

Intent: In order to better understand how to implement the GAPcare intervention to make it optimal for people with cognitive impairment and caregivers, the patient/caregiver may have suggestions.

9. If you were introducing the GAPcare intervention to your friends, what would you say about it?

10. Do you have any suggestions for how GAPcare could be improved? (probe, are there any other questions/concerns you might have about the intervention before deciding to participate?)

11. *To wrap up, is there anything else you'd like to share that I have not asked?*

Aim 1 Interview Guide for Caregiver Only

This interview agenda is a guide for the interviewers, but not a rigid script. We have added intent statements explaining the goals of a particular line of questioning for each major block of questions. Facilitators will use the agenda to ensure that all topic areas and probes are covered in the way we intended them to be covered. Questions may be added or deleted or altered in subsequent interviews as dictated by previously collected data (e.g. data saturation, emergent topics/themes) and in accordance with good qualitative methodology.

Intent: The intent of these interviews is to determine the perspectives of patients and caregivers after an emergency department visit for a fall to elicit barriers/facilitators, and ideas for how to facilitate post-fall care. We will review the participants' characteristics prior to the interview: age, race/ethnicity,

living situation, relationship to the caregiver. We will collect participants' reflections about the GAPcare intervention. Questions in this guide are intended for the caregiver.

Semi-structured interview protocol

Introduction:

Hello, my name is [name], and I am a [role] at the University of Colorado. Thank you for agreeing to meet with me today. We will be talking about [patient name]'s recent visit to the emergency department to learn more about how to improve care for patients after a fall. This interview should last about 30 minutes.

I'm just going to go over a few points from the consent form before we begin.

- You are being asked to be in this research study because [patient name] recently had a fall and visited the emergency department on [date].
- Every effort will be made to protect your privacy and confidentiality by not using your name on any research materials only a study ID will be used. All results will be submitted in aggregate form only and no names will be disclosed, so your responses will not be identifiable.
- This interview is voluntary. There are no right or wrong answers, and you may choose to answer or not answer any questions.
- If you have questions about the study, you can contact Dr. Elizabeth Goldberg at elizabeth.goldberg@cuanschutz.edu.
- You may have questions about your rights as someone in this study. If you have questions, you can call COMIRB (the responsible Institutional Review Board) at (303) 724-1055.
- I want to be sure I do not miss anything that you say, so I am asking your permission to record our conversation today. Do I have your permission to record?
- If you are ready, we will get started with the questions.

[State date and participant ID for the recording]

Barriers/Facilitators

Intent: The intent of this section is to understand participants' reactions to the planned GAPcare intervention, so as to understand what aspects of the intervention may need to be refined or specifically tailored to persons with cognitive impairment. Please provide them with the GAPcare handout to review prior to these questions.

1. The reason we are doing this study is because we want help patients who have had a fall and visit the emergency department. This handout shows the GAPcare intervention, which is extra care that patients like <patient's name> would get in the emergency department after having a fall. As you can see on the handout, with GAPcare a team of professionals, including physical therapists and pharmacists, would evaluate <patient's name> while you're waiting in the emergency department and then you would talk to this team to come up with a plan for how to prevent falls at home. What are your initial reactions to <patient's name> getting a special fall evaluation and treatment plan in the hospital?
2. Tell me how you think it would feel to talk to a physical therapist to figure out the reasons for <patient name's> fall and come up with a plan to support their care after they return home.

a. What about talking to a pharmacist about your current medications and how they could be changed to help prevent falls?

3. What concerns do you have about <patient's name> receiving care from a physical therapist, if any? (probe for Rationale)

4. Are there additional considerations that are unique to your culture or family that you think might impact the [patient's name] experience with GAPcare? (e.g., willingness to be involved, perceptions of receiving help from the healthcare team, ED procedures, followup procedures).

a. How do you think possible home treatments or remedies may work into this plan?

Acceptability

Intent: The intent of this section is to ask participants to describe their acceptability of the planned intervention. This section also assesses what support they need at home after a fall.

5. In your experience, would <patient's name> struggle with any of the following parts of GAPcare? These are some of the things they would have to do **in the emergency department**:

a. Speak with a pharmacist and respond to questions about their medication (e.g. what are their current medications, are they taking them, are they experiencing side effects, would they be open to modifying or stopping them with their doctor's permission?)

b. Participate in mobility exercises like getting up from a chair or walking with assistance with a physical therapist (probe for Rationale)

6. After visiting the emergency department, the plans that patients take home to help prevent falls can include a few different things. Now, I'd like to ask you about wat it might be like for **you to support** <patient's name> to do each of these activities **at home**.

a. Arrange a follow-up appointment with their primary care clinician. At the appointment, would they/you be able to talk to their doctor about the recommendations the pharmacist made in the hospital? (probe for Rationale)

b. Arrange an appointment with a physical therapist in an outpatient office or at home? (probe for Rationale)

c. Work with a physical therapist and do exercises at home and/or outpatient offices. What would it be like for you to help <patient's name> do that?

d. Help them to remove clutter and hazards away from areas where they walk?

e. Help them to use their cane/walker consistently when they are moving around the home?

f. Help them wear supportive shoe wear whenever they leave the bed?

g. Do you have any concerns about supporting <patient's name> at home after their fall?

h. What type of support would you need to help make sure <patient's name> is following recommendations like these, if any?

i. Would scheduled phone calls after the ED visit at 72 hours and one week help?

Adaptations (Cognitive/Cultural)

Intent: In order to better understand how to modify the GAPcare intervention to make it optimal for people with cognitive impairment and caregivers, especially those with diverse cultural traditions, we will ask questions about desired adaptations.

7. Many patients find it helpful to get reminders about what they are supposed to do once they get home. What do you think would help <patient's name> remember to follow recommendations for preventing falls at home? (E.g., Phone call reminders/magnet for fridge/follow-up letter/app or telehealth reminders/text messages)

a. What reminders would be helpful to you if you were helping <patient's name> follow a fall prevention plan?

b. For caregivers: If you typically do not attend ED visits with <patient's name> what type of handoff would you want to receive on the plan so you could act on it? (e.g. checklist, phone call, written document)

8. Based on what we have talked about so far, what role do you think the caregiver should play in the intervention? (e.g., accompanying patient to PT visits or to PCP visits to ensure GAPcare medication recommendations are incorporated, working with the patient to make medication changes to over the counter medications, etc.)

Implementability

Intent: In order to better understand how to implement the GAPcare intervention to make it optimal for people with cognitive impairment and caregivers, the patient/caregiver may have suggestions.

9. Do you have any suggestions for how GAPcare could be improved? (probe, are there any other questions/concerns you might have about the intervention before deciding to participate?)

10. *To wrap up, is there anything else you'd like to share that I have not asked?*

Aim 2 Interview Guide for Experts

This interview agenda is a guide for the interviewers, but not a rigid script. We have added intent statements explaining the goals of a particular line of questioning for each major block of questions. Facilitators will use the agenda to ensure that all topic areas and probes are covered in the way we intended them to be covered. Questions may be added or deleted or altered in subsequent interviews as dictated by previously collected data (e.g. data saturation, emergent topics/themes) and in accordance with good qualitative methodology.

The intent of these interviews is to determine perspectives from expert stakeholders about the modified GAPcareAD prototype protocol. We will obtain suggestions for how to enhance acceptability among clinicians and staff, overcome patient/caregiver identified barriers, aid communication with outpatient providers, informatics/technology approaches to automate screening, intervention content, implementing it with fidelity. We will review the participants' characteristics prior to the interview: age, race/ethnicity, title, profession, area of expertise, location. We will collect participants' reflections about the GAPcare intervention.

Semi-structured interview protocol

General consideration:

Intent: Prior to enrollment, participants will have provided verbal informed consent allowing us to collect their information on audio. We will explain how long the interview will last and what to expect; therefore, this section of the agenda is created to put the interviewee at ease and gather general impressions of their medication adherence.

1. [Show study brochure]. In GAPcare II, patients who come to the ED after a fall receive a pharmacy and physical therapy consult while they're waiting on their care. A pharmacist does medication therapy management and together with the patient and caregiver makes one to three recommendations for how to modify fall-risk increasing medications. Then a physical

therapist meets with the patient and caregiver and does a fall risk assessment, assesses reasons for the fall and provides recommendations including the need for outpatient PT, home PT, or transfer to a skilled nursing facility. Overall, how do you feel about offering a fall prevention team in the emergency department?

2. Tell me what regular care for falls in the emergency department looks like in your experience? (Probe: What's missing in regular care?)
3. How do care needs differ for persons living with dementia and their caregivers? (Probe: What are challenges to them getting the care they need? What has worked well?)
4. Can you describe your experiences with implementing clinical programs for patients with cognitive impairment and their caregivers, if applicable? What went well? What was challenging?

Adaptations to GAPcareAD

Intent: The intent of this section is to understand what adaptations experts would want.

1. [Cognitive Adaptations] The GAPcare intervention involves a pharmacist performing motivational interviewing surrounding reducing fall-risk increasing medications to the patient and caregiver. How do you think this would work for persons living with dementia?
2. [Cognitive Adaptations] The GAPcare intervention involves a physical therapist performing a fall risk assessment involving gait testing and measures of lower extremity strength and balance with the patient right after their fall. What are your initial reactions to this procedure when it is applied to persons living with dementia or cognitive impairment?
3. [Cognitive Adaptations] Persons living with dementia may struggle with executive function, attention, memory, and information processing. What changes would you make to GAPcare to accommodate for these differences in cognition?
4. [Cultural Adaptations] Think about the patient population in your local area. How would GAPcare need to be tailored for the communities your site serves?
5. [Affective-Motivational Adaptations] What do you think persons living with dementia would think of GAPcare? (Probe: What could we do to make GAPcare II more appealing for persons living with dementia?)

Fidelity

Intent: The intent of this section is to ask participants to describe their experiences with intervention modification and adapting it to a new setting/location/population with fidelity. This section also assesses what support or other changes may be needed to make it work within their local context.

6. What do you think it would be like to implement GAPcare at your local site? (probe for Rationale)
7. How do you think GAPcare would be received by ED clinicians at your site? (Probe: In your experience, what would make GAPcare easier for clinicians to accept? What do you think would make it hard for them to accept?
8. How would you feel about automating GAPcare recommendations in EPIC? We can automate a process for the GAPcare recommendations to get sent to the primary care clinicians through EPIC.

a. What challenges might arise with sending messages to primary care clinicians?

b. What modes would you suggest to increase uptake of the recommendations?

9. How would you examine fidelity at different sites? Site visits, review recordings of pharmacy/physical therapy interactions/have staff complete a fidelity checklist?

a. Did anything in particular influence your opinion of what method would be best?

10. What type of support/training do you think sites would need to implement GAPcare with fidelity?

Lessons learned

Intent: In order to better understand what content we should provide during implementation, we will ask a series of open ended closing questions.

11. Before you decide to implement GAPcare for persons living with dementia what are things you would want to know? What factors would you consider before deciding to use GAPcare?

12. If you were introducing GAPcare at a new study site, what would you say about it?

13. What advice do you have for the site principal investigators?

a. Any changes to the design that would help?

14. What other things should the site staff consider as they introduce this intervention?
